# Myosin Va and spermine synthase: partners in exosome transport

**DOI:** 10.1042/BSR20190326

**Published:** 2019-04-30

**Authors:** David J. Timson

**Affiliations:** School of Pharmacy and Biomolecular Sciences, University of Brighton, Huxley Building, Lewes Road, Brighton BN2 4GJ, U.K.

**Keywords:** cytoskeleton, exosome transport, Griscelli syndrome, myosin superfamily, polyamine synthesis, Snyder Robinson mental retardation syndrome

## Abstract

A recent paper in Bioscience Reports (BSR20182189) describes the discovery of an interaction between the motor protein myosin Va and the metabolic enzyme spermine synthase. Myosin Va is a molecular motor which plays a key role in vesicle transport. Mutations in the gene which encodes this protein are associated with Griscelli syndrome type 1 and the ‘dilute’ phenotype in animals. Spermine synthase catalyzes the conversion of spermidine to spermine. This largely cytoplasmic enzyme can also be localized to the soluble fraction in exosomes. Mutations in the spermine synthase gene are associated with Snyder Robinson mental retardation syndrome. The interaction between the two proteins was detected using the yeast two hybrid method and verified by microscale thermophoresis of recombinant proteins. Knockdown of the *MYO5A* gene reduced the expression of mRNA coding for spermine synthase. The amount of this transcript was also reduced in cells derived from a patient with Griscelli syndrome type 1. This suggests that, in addition to a direct physical interaction between the two proteins, myosin Va also modulates the transcription of the spermine synthase gene. The mechanism for this modulation is currently unknown. These findings have implications for Griscelli syndrome type 1 and Snyder Robinson mental retardation syndrome. They also suggest that interactions between myosin Va and soluble exosome proteins such as spermine synthase may be important in the mechanism of exosome transport.

## Myosin Va

Myosin is well known to undergraduates as the motor protein in muscles. However, the myosin superfamily encompasses a diverse set of motor proteins with roles in transport and motility [1,2]. All eukaryotes express at least one member of the myosin superfamily and over 20 classes of ‘unconventional’ myosins are recognized [3]. (In this context, ‘unconventional’ has come to mean ‘discovered after the muscle myosins’ as opposed to ‘different, rare or unusual’). All these proteins have at least one motor domain, which is reasonably well conserved structurally and functionally. Its role is to convert the chemical energy of ATP into mechanical energy. However, the domains attached to this motor vary widely and determine the role of the myosin. For example, several myosins have domains which enable their attachment to membranes facilitating their roles in membrane reorganization or the trafficking of membrane-bound organelles.

**Figure 1:**
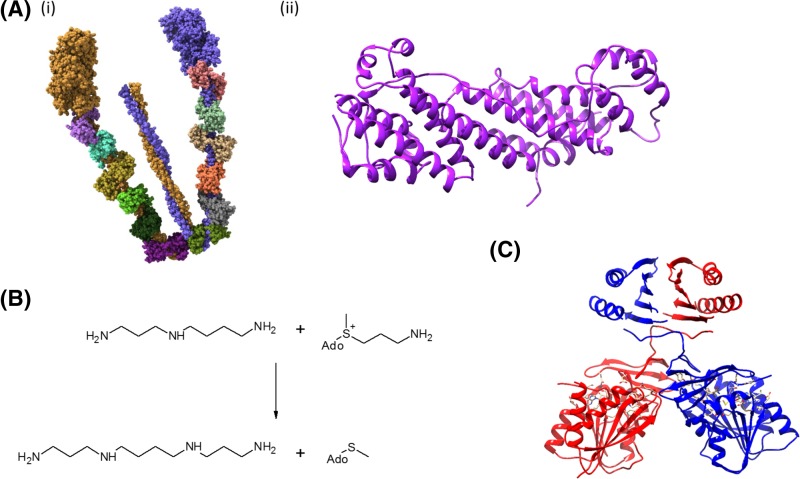
Structures of the proteins involved in this work (**A**)(i) Myosin 5a in the lower activity inhibited state (PDB: 2DFS [10]). The globular, motor domains are shown toward the top of the image. These form the N-terminal domains of the two heavy chains (purple and orange). The heavy chain then extends into a largely α-helical structure, which bends round in a U-shape, forming a coiled-coil region with the other heavy chain toward the C-terminus. Six calmodulin molecules (various colors) can be seen wrapped around this U-shaped region of each heavy chain. This structure lacks the GTD which is responsible for binding to the various ‘cargoes’ which are transported by the motor. (ii) The GTD from human myosin Va (PDB: 4J5L [9]). (**B**) The reaction catalyzed by spermine synthase. This reaction converts spermidine (*N*^1^-(3-aminopropyl)butane-1,4-diamine) to spermine (*N*^1^,*N*^4^-*bis*(3-aminopropyl)butane-1,4-diamine) using decarboxylated *S*-adenosylmethionine (dcAdoMet) as the donor of the 1-aminopropylgroup. (**C**) The structure of the spermine synthase dimer (PDB: 3C6M [36]). The two subunits of the dimer are shown in red and blue, with the N-terminal domain at the top of the image. Substrates can be seen bound to the C-terminal domain. Protein images were generated using UCSF Chimera, version 1.10.2 [53].

Myosin V is one of these ‘unconventional’ myosins [4]. It is found in most eukaryotes except plants. In humans, there are three isoforms: myosin Va, Vb and Vc [5]. Like myosin II (the family member responsible for muscle contraction), it has two non-covalently associated heavy chains and thus two motor domains. Myosin II is elongated with the two heavy chains held together with an extended coiled coil structure [6]. Between the motor domains and this coiled coil are extended α-helical sections around which are bound an essential light chain and the regulatory light chain. These light chains are structurally similar to calmodulin [7]. In contrast, each myosin V heavy chain consists of an N-terminal motor domain, an extended α-helical lever arm, a rod region which enables dimerization with the other heavy chain and a globular tail domain (GTD) [8,9]. The overall structure of myosin V is bent into a ‘W’ shape in the inhibited, low activity state and is elongated in the activated state (Figure 1A) [10]. Up to six calmodulins stabilize the lever arm and provide the means for calcium-dependent regulation of the motor [11]. The GTD is known to interact with a variety of other proteins and functions to link Myosin V to its various cargoes (for example, see [12–16]). The interaction of the GTD with membrane-tethered Rab GTPases is particularly important in the targetting of myosin V to vesicles [15,17–21].

Mutations in the myosin Va gene (*MYO5A*) are associated with the rare genetic disease, Griscelli syndrome type I (OMIM #214450) in humans and the ‘dilute’ phenotype in some other mammals [22–24]. Griscelli syndrome is associated with very pale skin and silvery-gray hair which appears in childhood. Patients typically have impaired brain function which results in developmental delays and cognitive disability. The ‘dilute’ phenotype describes lighter than average pigmentation or hair color in animals and is typically associated with substantially reduced lifespans [24,25]. A key function of myosin V is the transport of vesicles. For myosin V, these include vesicles containing pigments (melanosomes) [26–29]. Thus, impairment of myosin V function limits transport of pigments to the skin and hair surface resulting a paler or absent pigmentation. Myosin V is also required for fast axonal transport in nerve cells. The majority of this occurs on microtubules, with kinesins acting as the motor proteins. However, myosin V is the motor responsible for the actin-based part of the process [30,31]. This involvement in a key process in neuronal cells may partially explain the neurological defects associated with Griscelli syndrome.

## Spermine synthase

Spermine synthase (EC 2.5.1.22) catalyzes the conversion of the polyamine spermidine to spermine. Chemically, this involves the extension of the spermidine molecule to incorporate an aminopropyl group (Figure 1B). The cellular roles of spermine are not well defined, but the existence of a specific enzyme for its synthesis strongly suggest that it confers some form of evolutionary advantage [32]. Furthermore, mutations, which result in reduced activity of spermine synthase, are associated with the rare genetic disease, Snyder-Robinson mental retardation syndrome (OMIM #309583) [33,34]. This X-linked disease is characterized by cognitive and developmental abnormalities. The links between loss of enzyme activity and this pathology have not been fully elucidated. However, it has been shown that a build-up in spermidine, resulting from reduced rates of conversion to spermine, causes the production of toxic metabolites including N^1^-acetylspermidine and reactive aldehydes. These disrupt lysosomal and mitochondrial function and result in increased oxidative stress [35].

Human spermine synthase is homodimer. Each subunit has three domains, with an active site located in the C-terminal domain. Dimerization is mediated primarily by the N-terminal domain (Figure 1C) [36]. Spermine synthase is a cytosolic enzyme which is also known to be found in exosomes [37,38]. Exosomes are membrane-bound vesicles (50–100 nm in diameter) which are released from cells and are found in many biological fluids [39]. Their formation is tightly regulated, as is the selection of their protein, RNA and lipid contents [40–44]. They have been implicated in a number of diseases, including cancer and asthma [45,46]. Myosin V has been implicated in the transport of exosomes [47].

## Interaction

Dolce et al. set out to discover if myosin Va interacts with any of the soluble proteins commonly found in exosomes [48]. To do so, they conducted a yeast two hybrid screen using human myosin Va GTD as the ‘bait’ and a human cDNA library as the ‘prey’. While the yeast two hybrid screen is a well-established method to identify novel interactions, it is notorious for generating false positives (i.e., interactions which occur in the screen, but not *in vivo*) [49–51]. The authors took particular care to eliminate these false positives, for example, by removing zinc finger and heat shock proteins which commonly bind non-specifically to other proteins. Truncated (and most likely misfolded) parts of other proteins and translated portions of non-coding regions were also removed. This analysis identified four likely interaction partners for the GTD of myosin Va: spermine synthase, WD-repeat containing protein 48 (WDR48), tandem C2 domains nuclear protein (TC2N), and cold-shock domain containing protein E1 (CSDE1). Of these, the authors chose to focus on spermine synthase due to its role in neurodevelopment [52].

The interaction was verified by microscale thermophoresis using recombinant myosin Va GTD and spermine synthase. This estimated the dissociation constant (K_d_) at 3.5 µM. The two proteins colocalized near a subset of cytoplasmic vesicles in two different, cancer-derived cell lines. Knockdown of the *MYO5A* gene disrupted this localization of spermine synthase. This suggests that myosin Va helps spermine synthase localize to these structures. Silencing of *MYO5A* also resulted in a reduction in the expression of mRNA coding for spermine synthase and a small reduction of the amount of protein present in cells. In cells derived from a patient with Griscelli syndrome type 1, the amount of spermine synthase mRNA was also reduced [48]. This suggests the hypothesis that myosin Va may help regulate the expression of spermine synthase. This might be mediated by an interaction between the myosin Va protein and another (unidentified) protein which stimulates the synthesis of spermine synthase mRNA or a direct, stabilizing interaction between myosin Va and the mRNA. Alternatively, this regulation might be achieved through interactions between the mRNAs (or their precursors) which encode the two proteins.

That reduction in myosin Va protein levels result in down-regulation of spermine synthase expression suggests links between Griscelli syndrome type 1 and Snyder-Robinson mental retardation syndrome. Both diseases result in significant impairment to neuronal function which manifests as cognitive disability. Where mutations in the *MYO5A* gene cause decreased protein stability and thus reduced cellular concentrations, the amount of spermine synthase will also be reduced. This is likely to result in similar pathology to Snyder-Robinson mental retardation syndrome. It has yet to be determined whether or not spermine synthase levels reciprocally affect the expression of myosin Va. This interaction between myosin Va and spermine synthase may have other implications for both Griscelli syndrome type 1 and Snyder-Robinson mental retardation syndrome. Mutations in either gene may alter the corresponding protein such that the interaction has reduced affinity or is abolished altogether. This is likely to affect events downstream of the interactions, regardless of which gene was mutated.

The precise role of this interaction in exosome transport remains to be determined. It is not yet clear why interaction with a soluble (rather than membrane-bound) protein functions in this process. It is possible that myosin Va plays a role in trafficking spermine synthase to exosomes. Alternatively, myosin Va might recognize vesicles which already have spermine synthase associated with them. The role of spermine synthase in exosomes is also unclear. Nevertheless, the discovery of the interaction of myosin V and spermine synthase suggests new linkages between exosome transport and polyamine biosynthesis.

## Abbreviations

GTD: globular tail domain
